# Dengue: A Minireview

**DOI:** 10.3390/v12080829

**Published:** 2020-07-30

**Authors:** Harapan Harapan, Alice Michie, R. Tedjo Sasmono, Allison Imrie

**Affiliations:** 1 Medical Research Unit, School of Medicine, Universitas Syiah Kuala, Banda Aceh, Aceh 23111, Indonesia; 2 Department of Microbiology, School of Medicine, Universitas Syiah Kuala, Banda Aceh, Aceh 23111, Indonesia; 3 Tropical Disease Centre, School of Medicine, Universitas Syiah Kuala, Banda Aceh, Aceh 23111, Indonesia; 4 School of Biomedical Sciences, University of Western Australia, Nedlands, WA 6009, Australia; alice.michie@research.uwa.edu.au; 5 Eijkman Institute for Molecular Biology, Jakarta 10430, Indonesia; sasmono@eijkman.go.id

**Keywords:** dengue, epidemiology, pathogenesis, diagnosis, treatment, disease control

## Abstract

Dengue, caused by infection of any of four dengue virus serotypes (DENV-1 to DENV-4), is a mosquito-borne disease of major public health concern associated with significant morbidity, mortality, and economic cost, particularly in developing countries. Dengue incidence has increased 30-fold in the last 50 years and over 50% of the world’s population, in more than 100 countries, live in areas at risk of DENV infection. We reviews DENV biology, epidemiology, transmission dynamics including circulating serotypes and genotypes, the immune response, the pathogenesis of the disease as well as updated diagnostic methods, treatments, vector control and vaccine developments.

## 1. Introduction

Arboviruses (arthropod-borne viruses) are a taxonomically diverse group of viruses that are unique in their transmission between arthropod vectors and vertebrate hosts. They are classified according to antigenic relationships, morphology, and replicative mechanisms. Virus families that include arboviruses are Togaviridae, Flaviviridae, Bunyaviridae, Rhabdoviridae, Orthomyxoviridae, and Reoviridae [1,2]. The most clinically significant of the arboviruses belong to the genera *Flavivirus* (a member of Flaviviridae family) and *Alphavirus* (a member of Togaviridae family) [1]. There are five human epidemic arboviruses that have emerged or re-emerged in both hemispheres in recent decades: dengue virus (DENV), Zika virus (ZIKV), West Nile virus (WNV), yellow fever virus (YFV), and chikungunya virus (CHIKV) [3,4]. The first four viruses belong to the *Flavivirus* genus while CHIKV belongs to the *Alphavirus* genus. Among these viruses, DENV, CHIKV and ZIKV are considered the most epidemiologically important viruses globally [5,6]. It is estimated that approximately 3.9 billion people, living in more than 120 different countries, are at risk of infection with any of these three major arboviruses [7].

Dengue is the most important mosquito-borne viral disease in humans [8] and is caused by infection with any of four DENV serotypes (DENV-1 to DENV-4) [8]. DENV infection may result in a wide spectrum of clinical manifestations, from a mild flu-like syndrome, referred to as dengue fever (DF), to the potentially life-threatening dengue shock syndrome (DSS). The symptoms of DF include fever, nausea, vomiting, rash, aches and pains, while in DSS severe bleeding and shock can occur and, if untreated, mortality can be as high as 20% [9]. The previous World Health Organization (WHO) classification of dengue disease states, was composed of three disease categories: undifferentiated fever, DF and dengue hemorrhagic fever (DHF) [10]. DHF was then further classified into four severity grades, with grades III and IV defined as DSS. A revised WHO case classification was introduced in 2009 that replaced previous classifications with probable dengue, dengue without warning signs, dengue with warning signs and severe dengue. We review DENV biology; current epidemiology and transmission characteristics including circulating serotypes and genotypes; DENV-specific immune responses; disease pathogenesis; updated diagnostic methods; treatments and vaccine development.

## 2. Biology of DENV

### 2.1. The Structure of DENV

The mature DENV virion is characterized by a smooth surface that is approximately 50 nanometers (nm) in diameter, whereas the immature virion is 60 nm in diameter with a spiky surface [11]. The genome encodes three structural proteins (capsid (C, 100 amino acids (aa)), pre-membrane/membrane (prM/M, 75 aa (and envelope (E, 495 aa), and seven non-structural (NS) proteins (NS1, NS2A, NS2B, NS3, NS4A, NS4B and NS5) [11]. Structural proteins form the components of the DENV virion whereas non-structural proteins are involved in RNA replication [12]. A detailed description of the structural and non-structural proteins of DENV is presented in Table 1.

The C protein (12 kilodalton (kDa)), is a 100 aa residue, homodimeric protein that contains 26 basic aa residues and three acidic aa residues [13]. The C protein is crucial for nucleocapsid formation during the primary stages of DENV virion assembly [13] while the M protein plays an important role in the arrangement and maturation of the DENV particle [14]. The E protein is composed of three domains (domain I–III), with domain III responsible for receptor-binding activity [14]. The E protein is crucial for virus binding and fusion to host cell membrane [14].

The NS1 is a 45 kDa N-linked glycoprotein involved in the RNA replication complex. It is synthesized as a monomer and, after processing in endoplasmic reticulum and the trans-Golgi network, it is secreted as a hexameric lipoprotein particle into the extracellular space and blood [15]. The NS1 has been used as target of enzyme-linked immunosorbent assays (ELISA) and rapid immunochromographic assays [16]. Single-NS1-based testing has good diagnostic utility as a screening tool and to confirm DENV infection [17].

The NS2A is a ~22 kDa protein that also involves in the replication complex. Another NS protein, NS3 (618 amino acids), has multiple enzymatic functions including chymotrypsin-like serine protease activity, RNA helicase and RTPase/NTPase activity [11,12]. NS4A and NS4B, 16 kDa and 27 kDa, respectively, are integral membrane proteins and induce membrane alterations, important for DENV replication [11,12]. The NS5 protein (104 kDa), is the DENV methyltransferase–polymerase and has RNA-dependent RNA polymerase activity [12].

### 2.2. The Genome of DENV

The DENV genome has poor translational fidelity and a high mutation frequency (approximately 10^−3^–10^−5^ per nt per round of replication) [20]. The DENV genome, approximately 11 kilobases long, is a positive polarity single-stranded RNA (ssRNA) composed of a single, long open reading frame (ORF) flanked by two untranslated regions (5’-UTR and 3’-UTR) [12]. It has a type 1 cap structure (m7GpppAmpN2) at the 5’-UTR end and lacks a poly(A) tail at the 3’-UTR end [21,22]. Within the 5’-UTR (95–101-nt in length), there are six elements: stem-loop A (SLA), stem-loop B (SLB), 5’-upstream AUG region (5’-UAR), 5’-downstream AUG region (5’-DAR), C-coding region hairpin (cHP) and 5’-cyclization sequence (5’-CS). The last three elements are located within C protein-coding region [23]. SLA acts as the promoter for the viral RNA-dependent RNA polymerase (RdRp, e.i. NS5) [24]. Interaction between NS5 and SLA is necessary for viral replication [25]. SLB contains essential sequences for long range RNA–RNA interaction and genome replication [25]. Both the 5’-UAR and 3’-UAR are involved in genome cyclization [21]. The 5’-DAR is involved in RNA replication and DENV circularization [23]. The cHP (14 nucleotides (nt) long) is responsible for translation initiation from the C-start codon [23]. The 5’-CS mediates RNA–RNA interaction between the 5’- and 3’-ends of the viral genome and therefore is essential for genome cyclization [26].

The ORF, encoding both structural and non-structural proteins, is translated into a polyprotein that is processed co- and post-translationally by cellular and viral proteases, to produce ten mature viral proteins [23]. The N-terminal region of ORF encodes three structural proteins (C, prM/M, and E) and followed by seven NS proteins [11,23]. The 3’-UTR, approximately 450-nt long, is important for DENV replication and modulates viral growth and RNA synthesis in mammalian cells [27].

### 2.3. The Life Cycle of DENV

DENV replication involves several steps. First, DENV binds to cell-surface attachment molecules and receptors and is then internalized through receptor-mediated endocytosis. Several attachment molecules and receptors have been identified for DENV in mammalian cells including glycosaminoglycans, heat-shock proteins, neolactotetraosylceramide, CD14, C-type lectins such as dendritic cell-specific intracellular adhesion molecule-3 grabbing non-integrin (DC-SIGN) and the mannose receptors [19,28]. The E protein is involved in receptor-binding activity during viral entry [19].

Upon internalization, the low pH of the endosome triggers a conformational change in the E protein that mediates fusion of the viral and cellular membranes, allowing disassembly of the DENV virion [19,28]. The DENV nucleocapsid is released into the cytoplasm whereupon the virus uncoats and the DENV genome is released into the cytoplasm, where it is translated into a polyprotein that is processed by viral and cellular proteases [19]. The incoming positive-strand genome serves as mRNA for translation into a single polyprotein and, subsequently, as template for RNA synthesis. The polyprotein is then directed to the endoplasmic reticulum and is cleaved into the individual structural and NS proteins by host signalases and viral NS3 protein [12]. The newly synthesized RNA can be used for new rounds of translation or for encapsidation into new virions [21].

A negative-strand RNA intermediate serves as a template for new positive-strand viral RNA [19]. Viral RNA and proteins are then assembled into immature progeny virions at the endoplasmic reticulum membrane. Immature virions are transported through the secretory pathway (acidic environment of the trans-Golgi network) where the immature (spiky) virion transforms to mature (smooth) morphology [12,19]. During maturation, the pr peptide is cleaved from prM. M remains in the mature particle as a transmembrane protein. This process is mediated by a host-encoded furin protease [29]. In the final step, mature virions are released from host cells.

### 2.4. The Origin of DENV

There is strong evidence to suggest that DENV originated in non-human primates (sylvatic DENV) in Africa and Asia, with cross-species transfer to humans subsequently occurring independently with all four serotypes [30,31]. Sylvatic DENVs in Africa or Asia that utilize non-human primate hosts and forest-dwelling *Aedes* mosquito vectors are hypothesized to be ancestral for urban transmission [18]. Sylvatic DENVs are estimated to have emerged 1000 years ago, with transmission in human populations established as recently as the last few hundred years [20,32,33].

Malaysia is considered the sheltering area of the sylvatic ancestral DENV lineage for all serotypes [34,35]. A recent study suggests that DENV-1 evolved in Asia and later spread into Africa and the Americas [35]. The oldest DENV-1 isolate, the Mochizuki strain, was isolated in 1943 from Japan, with subsequent DENV-1 activity reported in the Americas in 1977 and in Africa in 1984 [34]. DENV-2 diverged from the sylvatic ancestor approximately 400–600 years ago [20,33]. This serotype was first reported in 1944 in Asia (Papua New Guinea and Indonesia), in 1964 in Africa (Nigeria) and in 1953 in the Americas (Republic of Trinidad and Tobago) [34]. DENV-3 was first reported in 1953 in Asia (the Philippines and Thailand), in 1963 in the Americas (Puerto Rico) and during 1984–1985 in Africa (Mozambique) [34]. DENV-4 was reported for the first time in Asia (in the Philippines and Thailand) in 1953 and in the Americas (Brazil, Cuba, Dominica, Puerto Rico, and the US Virgin Islands) in 1981 [34].

## 3. Epidemiology

### 3.1. Global Incidence and Mortality

The exact incidence of dengue is difficult to determine but estimates of the true number of annual dengue infections range from 284 to 528 million with 96 million of these being apparent cases [36]. Apparent cases are defined as all symptomatic infections, including those that are undetected by reporting systems. A report using 1636 country-years of case reports of dengue from 76 countries found a substantial increase in the incidence of dengue between 1990 and 2013, with the number of apparent cases more than doubling every decade, from 8.3 million in 1990 to 58.4 million in 2013 [36]. Among the cases that occurred in 2013, 10.5 million were treated in a hospital setting, 28.1 million were treated in an ambulatory health-care setting, and 19.7 million remained outside the health-care system [37]. The highest age-standardized incidence rates occurred in Southeast Asia, with an annual average of 34.3 cases per 1000 people [36].

Several studies have been conducted to determine the factors associated with the high incidence of dengue in certain regions. Several demographic, environmental, social, and ecological factors are associated with dengue incidence or outbreaks (Table 2).

Using 1780 country-years of mortality data from 130 countries, a recent study estimated that an average of 9221 people died from dengue per year between 1990 and 2013, with a peak of 11,302 in 2010. The highest mortality rate was observed in Southeast Asia [36]. In 2013, using data from 32 countries, another study estimated a ratio of one death per 5991 people with a global total of 13,586 dengue deaths, with 5838 and 7748 deaths occurring in children and in adults, respectively [37].

In 2013, an estimated 576,900 years of life were lost to premature mortality attributable to dengue, with a total of 566,000 years lived with disability (YLD) in which 89.9% of YLDs were from chronic fatigue, 7.8% of were from moderate acute infection and 2.9% were from severe acute infection [36]. In the same year, dengue was responsible for 1.14 million disability-adjusted life years (DALY) [36].

### 3.2. Global Economic Burden

Globally, the average cost per dengue case is approximately 84.73, 70.10, 51.16 and 12.94 USD for fatal cases, cases admitted to hospital, ambulatory cases, and cases outside the health-care sector, respectively [37]. The estimated total annual global aggregate cost of dengue in 2013 was 8.9 billion, USD equivalent to 1.56 USD per capita [37]. This estimate includes the cost of non-fatal cases admitted to hospital (4093 million USD), ambulatory non-fatal cases (2987 million USD), non-medical cases (752 million USD), and fatal cases (1055 million USD). However, a study in 2011 suggested a higher aggregate cost based on analysis of 95 million symptomatic cases occurring in 108 countries [54].

The aggregate annual economic burden of dengue was estimated as 2.1 billion USD annually between 2000 and 2007 among American countries and 950 million USD among Southeast Asian countries, with approximately 52–60% of these costs coming from productivity loss [55]. In 2012, the WHO estimated that Asia represents 75% of the global burden of dengue, costing Southeast Asia, alone, 1 billion USD annually [8].

### 3.3. Transmission of DENV

DENV is transmitted in both urban (human transmission cycle) and forested areas (sylvatic transmission cycle). Both of these transmission cycles are different ecologically and evolutionarily. Human transmission occurs in 128 countries where the main vectors are *Aedes (Ae). aegypti* and *Ae. albopictus* mosquitoes. In contrast, sylvatic transmission cycle takes place in the sylvan environments in Southeast Asia and West Africa where the main vectors are *Ae. luteocephalus, Ae. furcifer* and *Ae. taylori* [31]. In urban settings, DENV transmission occurs between humans, whereas in forested areas transmission occurs between non-human primates with occasional spillover into human populations [56]. Transovarial transmission (transmission of DENV vertically from mosquitoes to their offspring) is essential in maintaining both human and sylvatic transmission cycles during dry seasons or interepidemic periods [31]. Therefore, the eradication of DENV is challenging due to its transmission complexity. Non-vector transmission routes including blood transfusion, bone marrow transplant, and intrapartum and perinatal transmission have also been reported [57]; there is no evidence that DENV may be transmitted via semen as with Zika virus [58].

Enhanced globalization including rapid travel and trade have contributed to the rapid expansion of DENV [59]. Modern transport enables importation of dengue by overcoming natural barriers of travel time and geography, which had previously limited DENV expansion from endemic areas into non-endemic areas [60]. Indonesia has been consistently identified as a source of DENV infection in travelers and there is evidence that multiple DENV lineages have been introduced to the country over the years [61,62] making Indonesia a continuous hub for DENV transmission and mixing [61]. In Australia, more than 25% of imported dengue cases to Queensland were from Indonesia [63] and the majority of the dengue cases in Western Australia were imported by travelers returning from Bali [61]. In Taiwan, almost 24.8% of the imported dengue cases between 2011–2016 were also derived from Indonesia [64]

## 4. Serotypes, Genotypes and Lineages of DENV

DENV can be classified into four distinct serotypes (DENV-1, DENV-2, DENV-3 and DENV-4) that share approximately 65% amino acid sequence similarity. Each serotype includes several genotypes. A genotype is defined as a group of DENV isolates that have no more than 6% nucleotide sequence divergence [32]. Globally, DENV-1, DENV-2, DENV-3 and DENV-4 can be divided into five (I, II, III, IV, and V), six (Asian I, Asian II, Cosmopolitan, American, American/Asian and Sylvatic), four (I, II, III, and V) and four (I, II, III, Sylvatic) genotypes, respectively [65,66,67,68]. The geographical distribution of each genotype is presented in Table 3.

Different serotypes or different genotypes within a serotype may induce varied immune responses [56], and may vary in their ability to infect different target cells and, in turn, their capacity to cause the severe form of dengue [32]. For example, the American genotype of DENV-2 replicates with reduced efficiency in *Ae. aegypti* compared to the Asian genotype, and therefore is less transmissible [69]. This American genotype is also associated with less virulent strains [32]. Based on phylogenetic analysis of E gene sequences, each genotype can further be subdivided into multiple lineages [32]. Interestingly, the number of new lineages is growing [61,70,71].

### 4.1. Introduction and Replacement of Genotypes and Lineages

Molecular epidemiological studies may identify turnover (extinction and/or replacement) of DENV genotypes and lineages. In a certain geographical region, a particular genotype or lineage could emerge and persist for a number of years and then become extinct and be replaced with an entirely new genotype or lineage. Lineage turnover might be caused by a stochastic process, or arise due to variations in DENV fitness [72]. It has been reported that the introduction of new serotypes, genotypes and lineages into a region, and the subsequent replacement of endemic viruses, has occurred frequently for all serotypes [73,74,75,76,77,78,79,80,81,82], for example, for DENV-1 in Thailand in the early 2000s [77] and for DENV-2 in Vietnam in the early 2000s [78] as well as in America in the early 1990s [79]. DENV-3 lineage replacements were also identified in Thailand [80,81] and DENV-4 lineage replacement occurred in Puerto Rico during the 1980s and 1990s [82].

Emergent lineages have been observed in certain region. For example, a study in India identified a new lineage of DENV-3 during a 2006–2008 outbreak [71]. A new lineage of the DENV-2 Cosmopolitan genotype was identified in a 2011 outbreak in eastern India, with this lineage associated with increased severe dengue incidence [70]. A recent study also revealed the emergence of a new lineage of the DENV-2 Cosmopolitan genotype among Western Australian travelers returning from Indonesia [61].

### 4.2. Implication of Introduction and Replacement of Genotypes and Lineages

The introduction of new groups of viruses to populations with serological naivety has the potential to cause unprecedented outbreaks and has been associated with more severe dengue manifestations [70,78,79,83]. For example, introduction of the DENV-2 Asian/American genotype into the Caribbean in 1981 was associated with higher rates of the severe form of dengue [79]. Replacement of the DENV-2 Asian/American genotype with the DENV-2 Asian 1 genotype in Vietnam was attributed to higher viremia levels in patients [78]. A study in Peru found that lineage II of the DENV-2 Asian/American genotype was associated with a severe dengue outbreak in 2010 and 2011. This lineage was genetically distinct from lineage I of the DENV-2 Asian/American genotype which had previously circulated [83]. In India, introduction of a new lineage of the DENV-2 Cosmopolitan genotype was also associated with increased severe dengue manifestations [70]. Lineage I of the DENV-2 Asian genotype was associated with a higher DENV replication rate in human and mosquito cells compared to the DENV-2 Asian/American genotype [70]. This may be associated with higher transmission in both humans and vectors, potentially leading to disease outbreaks in regions where this lineage dominates [70].

In Indonesia, National Disease Surveillance System data reveal an increasing trend of dengue incidence in Indonesia in the last 50 years [84]. The incidence rates appear to be cyclic, peaking approximately every 6–8 years [85]. Over this 50-year timespan, serotype shifts, genotype displacement within DENV-1 and DENV-2 and genotype introductions of DENV-1 and DENV-3 from other countries occurred in Indonesia. Evidence suggests that these events were associated with increased incidence of dengue cases [85]. Therefore, it is critical to monitor the introduction of genotypes and lineages of DENV and to track the spread of genotypes and lineages that are known to be associated with severe forms of dengue.

Molecular epidemiology studies allow the movement of particular genotypes or lineages between regions to be traced and monitored. For example, phylogenetic analysis has shown that either Malaysia or Thailand was the most likely ancestral source of DENV-4, from where it then subsequently spread to other countries [86]. A phylogenetic study of 45 different geographical areas revealed that Indonesia and Thailand were the main sources of DENV-1 strains that circulate in other countries [35]. Furthermore, another study found that the continuing transmission of dengue in the Pacific (New Caledonia) was due to direct and multiple introductions of DENV from Asian countries [87].

## 5. Immune Response

Innate and adaptive immune responses are each critical in the defense against DENV infection. The innate immune system rapidly recognizes and responds to DENV, but does not provide a long-term or specific response. The innate immune response activates the complement system that helps the antibodies and leukocytes remove DENV [88]. The adaptive immune system, however, is more specific and involves cellular and humoral components. Both innate and adaptive immune responses to DENV infection contribute to the resolution of infection and play pivotal roles in protection from reinfection [88]. However, these responses may contribute to the enhancement of disease severity, causing severe dengue [88].

### 5.1. Innate Immunity

Langerhans cells, dermal cells, and interstitial dendritic cells are the initial targets for DENV infection [89]. Other cells such as monocytes, lymphocytes, Kupffer cells, alveolar macrophages, and endothelial cells are also potential target cells of DENV infection [89]. Upon inoculation of DENV, host pattern recognition receptors (PRRs) such as endosomal Toll-like receptors (TLRs), retinoic acid inducible gene I (RIG-I) and melanoma differentiation-associated gene 5 (MDA5) are responsible for sensing or recognizing the antigens of DENV [89]. The PPR molecules will activate two important families of transcriptional factors: the IFN regulatory factors (IRF) and the nuclear factor kappa B (NF-κB) that activate the production of IFN-α/β and inflammatory cytokines [89]. This will activate dendritic cells and establish an antiviral response. In vitro studies suggest that high level of cytokines are present in culture supernatants of DENV-infected cells [90,91]. An in vivo study also found a a high level of several cytokines such as TNF-α, IL-1β, IL-6, and IL-10 in sera of DENV-infected mice [92]. Higher plasma levels of IL-1β, IL-2, IL-4, IL-6, IL-7, IL-8, IL-10, IL-13, IL-18, TGF-1β, TNF-α, and IFN-γ have been found in patients with severe dengue [93]. These cytokines and chemokines can serve essential protective or detrimental roles during DENV infection [89]. It is believed that this “storm” of inflammatory cytokines and other inflammatory mediators acts on the endothelium and alters normal fluid barrier functions, leading to increased plasma leakage [89,93].

An important cytokine produced during DENV infection is IFN-γ. This cytokine is essential to the control of DENV replication and resistance to infection by controlling the production of nitric oxide [94]. Increased IFN-γ was associated with protection against fever and high viremia [95], with higher survival rates in DHF patients [96]. In contrast, other proinflammatory cytokines seem to play a pathologic role. For example, increased TNF-α level was associated with increased severity of dengue [97] and thrombocytopenia [98]. TNF-α can increase endothelial cell permeability in vitro [99] and high levels of TNF-α were correlated with endothelial cells apoptosis and hemorrhage in mouse model [100]. Another cytokine, IL-10, is correlated with platelet decay and may modulate coagulation activation in DENV-infected patients [93,101]. The macrophage migration inhibitory factor (MIF) also seems to have a deleterious role in DENV infection. Studies found the concentration of MIF is higher in severe dengue cases [98,102] and positively correlated with severity in DENV infection [103]. Other mediators and soluble factors such as monocyte chemoattractant protein 1 (MCP-1) [104], soluble vascular cell adhesion molecule 1 (VCAM-1) [105], and thrombomodulin [106] were also found to be increased in severe dengue.

During DENV infection, the activation of PRRs also mediates the production of chemokines that have dual protective and pathological effects. A study revealed that CXCL10 production and CXCR3 activation improved host resistance, as they compete with DENV for their cellular receptors and can therefore diminish DENV replication [96]. Other chemokines, CCL2 and CCL5, have reported associations with hypotension, thrombocytopenia and hemorrhagic shock, and hepatic dysfunction [89].

### 5.2. Cellular Immunity

The activation of T-cell responses is one of the most important components in DENV-specific immunity. The principal target for both of CD4^+^ and CD8^+^ T cells is located within NS3 [107]. DENV-specific T cells enhance DENV clearance by inducing infected-cell lysis and enhancing production of a wide range of cytokines [107]. The efficiency of clearance is dependent on the avidity of the T-cell receptor (TCR) for the HLA-peptide complex [93].

However, T cells may cause immunopathology during DENV infection, in a phenomenon called original antigenic sin [108]. This phenomenon is defined as the dominance of T cell responses mounted against a previously infecting serotype, over the current infecting serotype [109]. During a primary infection, T cells and cross-reactive memory T cells are produced. Upon secondary infection with a heterologous serotype, highly cross-reactive CD8^+^ T cells with a high avidity for the secondary DENV infection are activated massively and induce high production of proinflammatory cytokines. In addition, low-avidity cross-reactive CD8^+^ T cells are expanded. Cross-reactive CD8^+^ T cells against heterologous serotypes may lose their cytolytic activity [109]. This may delay DENV clearance, prolong activation of cross-reactive CD8^+^ T cells and induce a high level of proinflammatory cytokines and other soluble factors. Together, these factors affect vascular permeability, leading to a higher incidence of severe dengue [93,107].

### 5.3. Humoral Immunity

The humoral immune response is essential for controlling DENV infection. The principle DENV epitopes, as targets for the antibody response to DENV, are within the E, NS1 and pre-M proteins [107]. The E protein induces the production of antibodies that have pivotal roles in DENV neutralization, while the NSI protein activates antibody-dependent cellular cytotoxicity and complement-dependent lysis of infected cells [107]. Incomplete cleavage of pre-M protein also induces the pre-M protein-specific antibody that is highly serotype cross-reactive [107].

A report documented that infection with one serotype gave long-lasting protection to that specific serotype (homotypic immunity) [110]. Phase II [111,112,113,114,115] and phase III trials [116,117] of the Sanofi Pasteur dengue vaccine provide confirmation of the protective response offered by the anti-dengue neutralizing antibody. However, DENV sub-neutralizing antibodies may also detrimentally enhance DENV infection of the cell targets, a mechanism known as antibody-dependent enhancement (ADE) [118].

The antibody response to DENV differs depending on the immune status of the host. During a primary infection, a primary antibody response is detectable in 50% of hospitalized patients by 3–5 days after the onset of illness and increases to 80% by day 5 and 99% by day 10 [16,119]. In one study, nearly all patients (93%) developed detectable IgM 6 to 10 days after the onset of fever, and 99% of patients screened between 10 and 20 days after onset had detectable IgM [120]. Anti-dengue IgM levels peak about two weeks after the onset of symptoms and then decline generally to undetectable levels over 2–3 months. Anti-dengue serum IgG is generally detectable at low titer by the end of the first week of illness, increasing slowly thereafter and may be detectable for the lifespan of the patient [119]. During a secondary dengue infection, antibody levels rise rapidly which are broadly cross-reactive with many flaviviruses. The IgG is the dominant immunoglobulin isotype, which is detectable at high levels, even in the acute phase, and persists for periods lasting from 10 months to life [119].

## 6. Pathogenesis

Dengue pathogenesis is influenced by both viral and host factors that remain incompletely understood. Severe dengue may occur in those experiencing a secondary infection with heterotypic strain of DENV and in infants who are born to dengue-immune mothers with primary anti-DENV antibody responses [121,122,123,124]. This phenomenon, ADE, may be explained by two concurrent processes. First, during primary infection, serotype cross-reactive and sub-neutralizing antibodies are produced. Second, during secondary infection with a heterologous serotype, sub-neutralizing antibodies produced during the primary infection bind to the second infecting DENV and these antibody-virus complexes are internalized into target cells via Fc gamma receptor (FcγR) resulting in enhanced infection [93,125]. The exact mechanism of ADE remains unclear, but evidence suggests that it is associated with both increased DENV infectivity and the suppression of host immune responses. Once the DENV antibody is below the neutralization threshold, the number of infected target cells is increased (extrinsic ADE), enhancing DENV production [126,127,128]. In addition, non-neutralizing antibodies also alter immune responses and cellular functions, leading to an increase in progeny DENV produced per infected cell, or ‘burst size’ (intrinsic ADE) [129,130,131,132]. Both extrinsic and intrinsic mechanisms have been proposed as the main mechanisms involved in ADE.

The ADE phenomenon is supported both by animal models [133,134] and epidemiological studies [123,135,136,137,138,139,140]. Passively transferring DENV monoclonal antibody into an animal model resulted in a significant enhancement of viremia and clinical dengue manifestations [133,134]. Epidemiological studies revealed that the incidence of severe forms of dengue was higher during secondary DENV infection [123,135,136,137,139,140]. A study in dengue epidemic areas of Cuba found that infection of DENV-1 followed by DENV-2 or DENV-3 was associated with higher incidence of severe dengue [24]. Prospective cohort studies in Asia and Latin America have identified secondary infection as an epidemiological risk factor for severe dengue [135,136,141,142,143,144]. The interval between sequential infections is important. The longer the interval between two sequential DENV infections (approximately beyond two years), the higher the proportion of severe dengue cases during secondary heterotypic DENV infection [145,146]. Interestingly, a study revealed that the vast majority of tertiary or quaternary infections are clinically silent or very mild [147].

During ADE, the binding between virion and target cells is facilitated by two types of FcγR (I and IIa) that are expressed on monocytes, macrophages and dendritic cells [132]. ADE enhances the membrane fusion efficiency of the DENV virion into target cells [148]. Membrane fusion then initiates an aggravating cascade, leading to enhancement of infection, and promotes intrinsic ADE mechanisms leading to enhanced DENV production [148,149].

DENV can suppresses the innate immune response (intrinsic ADE) during ADE, and can therefore facilitate longer survival of infected cells and thus increased total DENV output [126,130,132,150]. Studies found that ADE enhanced DENV burst size in a range between 5- to 7-fold in primary macrophages and monocytes [126,127,128] and increased the number of virions production 10-fold in human PBMCs [151], 1500-fold in monocytes, 3000-fold in mature dendritic cells, and 2000-fold in monocyte-derived-macrophages [127]. Mechanisms have been proposed to explain the intrinsic role of enhancing antibodies in increasing DENV production. It is postulated that ADE alters the production of type 1 interferons (INF), such as IFN-β, and anti-inflammatory cytokines by several mechanisms. ADE suppresses mitochondria antiviral protein-mediated signaling [152] and decreases the expression of PRRs, RIG-1 and MDA5 [153], therefore downregulating IFN-β expression. DENV sub-neutralizing antibodies also inhibit the Toll-like receptor (TLR) signaling system [131]. ADE also stimulates the expression of the anti-inflammatory cytokines including IL-10 [150] leading to the suppression type 1 IFN signaling [129]. In addition, ADE inhibits FcγR signaling and therefore reduces early antiviral responses associated with type 1 IFN [154]. ADE suppresses the expression and activity of inducible nitric oxide synthase 2 (NOS2) [153] by disrupting the NOS2 gene transcription factor, IRF-1 [152]. NOS2-derived nitric oxide (NO) is an effector of the innate immune system and is involved in the control of pathogens [153,155].

Not all studies support the notion that severe dengue is associated with secondary infection of heterologous serotypes. For example, a study in an animal model found that infection of DENV-1 or DENV-4 followed by a DENV-3 infection did not increase dengue severity [105]. Another study found that the incidence of DHF between vaccinated and unvaccinated children with an experimental live-attenuated DENV vaccine was no different [156].

A large number of other hypotheses have been proposed to explain the pathogenesis of severe dengue [121]. It has been suggested that increased vascular permeability in severe dengue is mediated by antigen-antibody-complement complexes [121,157,158]. During a second heterotypic dengue infection, the circulating DENV antigens and anamnestic IgG dengue antibodies activate complement resulting in a reduced level of C3 and increased levels of C3a and C5a anaphylatoxins [157,158]. This excessive complement activation at endothelial surfaces contributes to vascular leakage in severe dengue [159]. There is a correlation between the complement system and NS1 in which NS1 is an important trigger for complement activation [160]. Binding of heterotypic antibodies to NS1 results in complement activation [159,161].

The original antigenic sin hypothesis may also explain dengue pathogenesis. Studies revealed that recognition between different DENV peptides was associated with reduced CD8^+^ T cells cytolytic potential without reducing cytokine production [109,162,163]. This pathogenic heterologous T-cell response results in cytokines and chemokines production (cytokine storm) that will increase vascular permeability [93,121]. Increased permeability of the vascular endothelium, enables leakage from the intravascular space [164]. Higher concentrations of cytokines such as IFN-γ, TNF-α and IL-10 were observed in the sera of patients with severe dengue in Cuba [165], India [166] and Vietnam [167]. Increased IL-10 levels correlate with reduced levels of platelets and reduced platelet function, contributing to the development of bleeding complications [168]. TNF-α promotes increased endothelial permeability [169].

Host factors may also contribute to the pathogenesis and clinical outcomes of dengue [170]. Our previous review provides evidence that single-nucleotide polymorphisms (SNPs) both in human leukocyte antigen (HLA) [171] and non-HLA [172] genes are associated with uptake of virus and the severity of dengue disease. SNPSs in major histocompatibility complex (MHC) class I chain-related protein B (MICB) and phospholipase C epsilon 1 (PLCE1) genes were associated DSS [173,174]. Polymorphisms in TNF-α, FcγRII, cytotoxic T-lymphocyte antigen 4 (CTLA-4), transforming growth factor (TGF)-β1, human platelet antigens (HPA), dendritic cell-specific ICAM3-grabbing non-integrin, transporters associated with antigen processing (TAP), and Janus kinase 1 (JAK1) genes were associated with severe forms of dengue [172]. Altogether these indicate a pivotal role of host factors in the pathogenesis of dengue.

In addition to the characteristic DENV- associated hemorrhaging, neurological complications such as encephalitis and encephalopathy are also of major concern for dengue patients. Although the mechanisms of neuropathogenesis are still not well understood, direct invasion of the central nervous system by DENV and metabolic alterations are likely the most important mechanism for encephalitis and encephalopathy [175,176]. The detection of DENV or DENV antigens among patients admitted with dengue encephalitis indicates that direct virus invasion of the central nervous system is the main neuropathogenesis for the mechanism of encephalitis [177,178]. Encephalopathy is associated with several factors including metabolic disturbances, liver failure, renal failure, systemic or cerebral hemorrhages or acute cerebral edema [177,179].

## 7. Clinical Features

Dengue is a systemic and dynamic disease with a wide clinical spectrum ranging from mild to severe clinical manifestations. In general, following an incubation period, the illness may be classified into the three phases: febrile, critical and recovery [119]. During the febrile phase, patients develop a sudden high-grade fever that lasts for 2–7 days that is often accompanied with facial flushing, skin erythema, body aches, myalgia, arthralgia, headache, severe retro-orbital pain and other symptoms such as anorexia, nausea, vomiting, sore throat, injected pharynx and conjunctivitis. In the early febrile phase, it is difficult to distinguish these signs from other non-dengue febrile diseases and it is indistinguishable between severe and non-severe dengue cases [119].

The second critical phase occurs around the time of defervescence (the lessening of fever) typically on days 3–7 [119,180]. Defervescence is temporary, lasting for around 48 h, and is associated with an increased propensity for capillary leakage and hemorrhage. At this stage, patients without increased capillary permeability will improve, while those with increased capillary permeability may become worse as a result of plasma leakage, and their conditions may become life-threatening [119]. Not all patients infected with DENV will develop severe dengue. In Singapore, only 4.1% out of 3186 dengue cases were diagnosed with severe dengue with 5.4% CFR. The infecting DENV serotype and genotype can significantly contribute to disease severity. Other factors have been proposed, such as those listed in Table 4, including various clinical findings, patient demographics and the type of infection (primary vs. secondary). A meta-analysis found that DENV-3 from the Southeast Asia region had the greatest percentage of severe cases in primary infection. Other serotypes from this region, as well as DENV-2 and DENV-3 from non-Southeast Asian regions, exhibited the greatest percentage of severe cases in secondary infection [181]. Moreover, in Southeast Asia, DENV-2 and DENV-4 are more highly associated with DSS, while DENV-3 and DENV-4 were more highly associated with DHF [181].

Prolonged shock may lead to organ impairment, metabolic acidosis and disseminated intravascular coagulation. This in turn leads to severe hemorrhage [119]. Atypical clinical features of dengue may also develop without obvious plasma leakage or shock including encephalitis, myocarditis, hepatitis, pancreatitis, retinitis and acute respiratory distress syndrome [119,180]. Two possible outcomes in this phase are either death or proceeding to recovery phase if the patient survives the first 24–48-h critical phase. Some factors are associated with high dengue mortality (Table 5).

In the final, recovery phase, a gradual reabsorption of extravascular compartment fluid occurs in the following 48–72 h [119]. There is an improvement in general well-being, the return of appetite and the stabilization of hemodynamic status. Persistent symptoms such as headache, retro-ocular pain, insomnia, alopecia, myalgia, arthralgia, asthenia, anorexia, dizziness, nausea, vomiting and itch are common, and these symptoms are associated with alterations in some immunological parameters [213]. In addition, a study also found that neurological complications including encephalopathy, myelopathy, myositis and peripheral neuropathy are common post-dengue infection [214].

## 8. Diagnosis

Diagnosis of dengue based on clinical symptoms alone is unreliable, due to the wide spectrum of non-specific symptoms during febrile illness. Specific and sensitive diagnostic tools are available that are suitable during certain phases of disease. During early infection (<5 days), dengue may be diagnosed by virus isolation, RNA detection (NAAT: nucleic acid amplification tests) or detection of antigens such as NS1. Following this period (>5 days after infection), DENV RNA and antigens may no longer be detectable, as only viremia has subsided and antibody responses are mounted. Specific antibody detection using serological methods (detection of IgM or IgG) is appropriate at this stage [119]. The NS1 antigen may be detected in some patients for several days after defervescence [119].

### 8.1. Virus Isolation

Virus isolation is very specific and can confirm a DENV diagnosis. DENV can be isolated by the inoculation of clinical specimen onto cell lines such the mosquito cell line, C6/36 (*Ae. albopictus*) or onto mammalian cell lines such as Vero (African green monkey kidney), LLCMK2 (Monkey Rhesus kidney) and BHK21 (baby hamster kidney) [16]. Clinical specimens used for viral isolation may be whole blood, serum, plasma or homogenized tissue (most often in fatal cases) [16]. After inoculation and an incubation phase, a confirmation assay such as immunofluorescence assay or reverse transcriptase polymerase chain reaction (RT-PCR) is required. There are several practical limitations virus isolation: (a) it is tedious and requires at least 7 days for incubation and confirmational testing; (b) it requires well-established lab facilities with well-trained personnel; (c) the window period for sample collection is limited to the acute phase of infection; and (d) a low level of DENV viremia is not suitable for virus culture [215].

### 8.2. Nucleic Acid Amplification Tests

Nucleic acid amplification tests may be used to diagnose dengue during the acute phase of infection (<5 days) and can detect DENV RNA in a clinical specimen within 24–48 h after infection. Techniques include RT-PCR, real-time RT-PCR, or isothermal amplification methods. RT-PCR may be conducted using the nested RT-PCR method [216], one-step multiplex RT-PCR (a combination of the four serotype-specific oligonucleotide primers in a single reaction tube) [217] or the one-step pan-flaviviruses quantitative RT-PCR assay [218,219]. The sensitivity of the RT-PCR methods varies from 80% to 100% and depends on the genome region targeted by primers, the approach used to amplify or detect the PCR products, and the method employed for serotyping [119]. Multiplex real-time RT-PCR assay is faster and is able to determine the viral titer in a clinical sample [137]. However, this test requires expensive equipment and reagents and must be performed by experienced technicians [119]. One-step pan-flaviviruses quantitative RT-PCR assay have equivalent characteristics compared to species-specific RT-PCR assay [218,219].

### 8.3. Detection of Antigens

New ELISA and rapid immunochromographic (IC) assays that target NS1 have been able to detect primary and secondary DENV infection up to 9 days after the onset of illness. A meta-analysis including 30 studies from various countries found that the NS1 ELISA kit from Panbio had 66% sensitivity and 99% specificity, while the NS1 ELISA kit from Platelia had 74% sensitivity and 99% specificity [220]. Another meta-analysis found that the IC assay based on NS1 antigen detection had slightly higher sensitivity compared to ELISA (71% vs. 67%) [17]. In general, NS1-based assays have good diagnostic utility, for both screening for and confirming DENV infection [17]. However, there are some issues related to this assay. The sensitivity of NS1-based tests is lower during secondary infections [220,221]. In addition, the sensitivity is lower for DENV-4 and DENV-2 (compared to DENV-1) [17,220] and is slightly lower in samples from Southeast Asia and Oceania [220].

### 8.4. Serological Tests

Serological tests such as hemagglutination inhibition (HI) assay and ELISA to detect IgM and IgG are more widely used to diagnose dengue in developing countries, as they are simple to perform, relatively inexpensive and the specimens required are stable at room temperature. The HI assay is based on the ability of E protein to agglutinate red blood cells (RBCs). Anti-DENV antibodies present in sera inhibit this agglutination and the extent of this inhibition is measured in the HI assay [119]. The HI assay has some limitations that make it is impractical, such as: (a) each serotype requires a different optimal pH of RBCs and therefore requires the use of multiple pH buffers; (b) it is unable to discriminate infections either between DENV and other related *Flaviviruses* or between immunoglobulin isotypes (IgM vs. IgG); and (c) it may require chemical and heating pre-treatment to remove nonspecific inhibitors in the clinical specimen [119,215]. Therefore, this assay has been largely replaced by ELISA-based methods for the detection of dengue specific IgM and IgG. The sensitivity and specificity of IgM detection ELISA, using sera collected five days or more after the onset of fever is ~90% and 98%, respectively [16]. The sensitivity and specificity of IgM-based assays is strongly influenced by the quality of the antigen used and can vary greatly between commercially available kits, as evaluated by a recent study [16,222].

Another antibody detection assay is the plaque reduction neutralizing test (PRNT). This assay measures neutralizing antibodies that inhibits DENV infection and therefore provides greater specificity in distinguishing antibodies to DENV from other cross-reactive flavivirus antibodies [223]. However, this assay is labor-intensive, has low throughput and is time consuming. For these reasons, the PRNT is not used in routine diagnostics. To overcome these limitations, a new generation of PRNT-based methods such as enzyme-linked immunosorbent spot microneutralization assay [224] and ELISA-based microneutralization test [225] have been developed.

## 9. Treatment

There are currently no specific treatments or cures for dengue. Current treatment options are supportive, and aim to limit the complications and severity of symptoms. Fluid therapy is one such key therapy in dengue management. Oral fluid replacement is sufficient for DF; in severe dengue, however, intravenous fluid replacement should be performed for shock prevention [226]. Specific details for the management of different severities of dengue are provided in the most recent WHO guidelines [119].

There are currently no specific drugs available or approved by the US Food and Drug Administration (FDA) for use against dengue. Several candidate anti-dengue therapeutic agents (that target viral or host components) have been tested in clinical trials such as carbazochrome sodium sulfonate for preventing capillary leakage [227], oral prednisolone as an anti-inflammatory agent [228] and lovastatin (statin) as an anti-DENV and anti-inflammatory at the endothelium [229]. Treatments to reduce severe bleeding or shorten the time to cessation of bleeding, such as single platelet donations [230] or recombinant human (rh) IL-11 [231], have been tested in small-scale trials. Other anti-DENV agents such as chloroquine [232], balapiravir (nucleoside analogue and a polymerase inhibitor) [233] and celgosivir (glucosidase I inhibitor) [234] have been also tested in trials. Table 6 presents a summary of clinical trials that have been conducted for dengue treatment.

Progress towards the development of effective therapeutics has been slow in recent years [170] and there is still an unmet need for an effective anti-dengue drug [235]. The ideal characteristics of a dengue therapeutic would have pan-serotype activity, able to rapidly resolve symptoms, be well tolerated with minimal toxicity, be readily distributable at large-scale, have minimal interaction with other drugs and be tolerable for adults, children, infants, pregnant women and patients with co-morbidities [235]. Several small components have been developed such as viral entry or fusion inhibitors, replication and transcription inhibitors, methyltransferase inhibitors, helicase inhibitors, protease inhibitors, and an NS4B inhibitor [235] as well as monoclonal antibodies [236]. There are several challenges in making this ideal anti-dengue therapy [235]. For example, it has been difficult to identify an inhibitor that is active against all four DENV serotypes given the high amino acid sequence variation (30–35%) between these groups [237]. It is challenging to mount antibodies that are equally protective against all serotypes and the administration should trough intravenous [237]. In addition, no accurate animal model exists that mimics human DENV pathogenesis [238] which hampers progress towards a safe and effective therapeutic.

## 10. Vector Control and Prevention

Vector control remains the primary method in the prevention of dengue infections. This can be achieved through environmental interventions, chemical control using insecticides and larvicides, and biological control. Environmental interventions involves the reduction in or elimination of natural and man-made vector breeding sites, such as containers and poorly managed waste facilities. Although environmental interventions are considered as safe strategies, a current systematic review and meta-analysis found the effect of these interventions on reducing larval populations was weak [241]. Chemical control involving insecticides has been utilized in dengue vector control for many decades with vector resistance reported in several regions [242]. Nevertheless, this strategy is still the mainstay vector control during dengue outbreaks [243]. A systematic review found that both indoor application of insecticides, indoor space spraying (ISS) and indoor residual spraying (IRS), had a very good effect in reducing adult mosquitoes but the effects on larvae abundance were fewer [244]. Other chemical control strategies, such as insecticide-treated windows, door screens or curtains, insecticide or larvicide-treated water container covers and insecticide-treated bed nets, have varied impacts on vector abundance as measured by the container index (CI) and Breteau index (BI) [245]. In recent years, modern, integrated vector management approaches using novel biological control approaches such as paratransgenesis [246,247,248,249,250,251,252,253,254], sterile insect techniques [246,247,255] and the production of genetically modified vectors [246,247,256,257,258] have been also developed.

Behavioral protective measures for limiting human exposure to mosquitoes include using insect repellent, wearing full coverage clothing, and the use of mosquito bed nets and window screens [259,260,261]. However, most of these strategies are compliance- and community involvement-dependent and are hindered by poor compliance and the lack of active community involvement [262,263,264].

## 11. Vaccines

Government and policy-makers in dengue hyperendemic regions start to consider the possible advantages of dengue prevention program using vaccination strategies [265]. The dengue vaccine, CYD-TDV from Sanofi Pasteur, has recently been approved in some countries. Phase II trials of CYD-TDV were conducted in Latin American countries such as Brazil, Colombia, Honduras, Mexico, Puerto Rico and Peru [111,112,114] as well as Singapore [113], and Thailand [115]. A meta-analysis of 6678 samples (4586 vaccinees and 2092 placebo group) found greater safety in the CYD-TDV group compared to the placebo group [266]. Phase III trials for CYD-TDV were conducted in Asia [117] and Latin America [267] that assessed the long term-efficacy and safety of CYD-TDV [116]. In general, trials in Latin America found the efficacy of CYD-TDV was 60.8% [267] and 56.5% in Asia [117].

Tetravalent dengue vaccine (TDV), previously named DENVax, is a chimeric dengue-2 PDK-53-based tetravalent vaccine developed by Takeda Vaccines Inc [268]. Two Phase I studies that evaluated the safety and immunogenicity of TDV in *Flavivirus*-naïve adults have been conducted [269,270]. There were no vaccine-associated adverse effects observed in these trials and it was found that TVD induced a high viremia in dengue-naïve adults rapidly. TVD induced production of NAb to all four DENV serotypes in dengue-naïve healthy adults [269,270]. In addition, the second dose was found to result in seroconversion in 100% of tested individuals for DENV-1, 94–100% for DENV-2, 82–100% for DENV-3 and 46–78% for DENV-4, 30 days after the second intradermal or subcutaneous dose [269,270,271]. A Phase II trial to evaluate the safety and immunogenicity of TDV in dengue-exposed populations in four dengue-endemic countries (Puerto Rico, Colombia, Singapore and Thailand), in both adults and children has been completed [272]. This study found that injection site pain, itching and erythema were the only solicited adverse events. In addition, TVD induced anti-DENV seropositivity in >95% of people against DENV-1–3 and 72.7–100% against DENV-4. Geometric mean NAb titers (GMTs) of DENV-1 ranged from 582–1187; DENV-2 from 582–1187; DENV-3 from 196–630 and DENV-4, from 41–210 [272]. A Phase III clinical trial in Asia and Latin America found that the overall TVD efficacy was 80.9% in preventing virologically confirmed dengue caused by any DENV serotype [273].

Another dengue vaccine candidate is TV003/TV005, a live attenuated tetravalent dengue vaccine that has been developed. This vaccine involves different monovalent vaccine candidates, produced by introducing a 30-nt deletion in the 3′ untranslated region (3’ UTR) into a wild type DENV, that are incorporated into tetravalent dengue vaccine. In a randomized, double-blind clinical trial, different monovalent vaccine candidates were combined into five different tetravalent admixtures (TV001–TV005) and were evaluated in 113 *Flavivirus*-naive adults [274]. This study found that there was no significant difference in the incidence of adverse events between vaccines and placebo-recipients, other than rash [274]. TV003 induced a trivalent or greater antibody response in 90% of *Flavivirus*-naive vaccinees and TV003 elicited the most balanced and broad antibody response, with 45% of participants seroconverting to all four DENV serotypes after only 1 dose. Seroconversion rates were high for DENV-1, DENV-3, and DENV-4 serotypes (85–100%) but low for DENV-2 (50%) [274]. Another Phase I trial was conducted among 168 *Flavivirus*-naive adults to investigate the safety and immunogenicity of TV003, compared with those of a second tetravalent vaccine with an enhanced DENV-2 component (TV005), and to evaluate the benefit of a booster dose at 6 months [275]. A single dose of TV005 elicited a tetravalent response in 90% of participants by 3 months post-vaccination and induced seroconversion rates to DENV-1–4 of 92%, 97%, 97% and 97%, respectively [275]. A single dose of TV005 induced 74% of tetravalent response and induced seroconversion rates to DENV-1–4 of 92%, 76%, 97%, and 100%, respectively [275]. The safety and immunogenicity of TV003 and TV005 are currently being evaluated in the Phase II trials in Thailand and Brazil [276].

The nucleic acid-based dengue vaccine, D1ME^100^, against each of the four serotypes has been developed by Naval Medical Research Center, United States. D1ME^100^ is a monovalent plasmid DNA vaccine expressing the prM and envelope (E) genes of DENV-1 virus [277]. Studies found that DENV-1, DENV-2 and DENV-3 DNA vaccines can elicit anti-dengue NAb in mice and non-human primates [278,279,280] and the prototype DENV-1 vaccine tested in rhesus macaques and *Aotus* monkeys provided 80–95% protection against live virus challenge [278,281,282]. A Phase I clinical trial, involving 22 healthy *Flavivirus*-naïve adults administered three intramuscular injections (0, 1, and 5 months) found that the most commonly reported adverse effects were local mild pain or tenderness, local mild swelling, muscle pain and fatigue [277]. Approximately 40% of subjects in the high dose group and none in the low dose group developed detectable anti-dengue NAb [277]. Two other dengue vaccines, TDENV PIV (a tetravalent purified inactivated vaccine) and V180, a recombinant subunit vaccine based on the DENV wild type prM and truncated E protein (DEN-80E) via expression in the Drosophila S2 cell expression system, are currently being evaluated in Phase I clinical trials [276].

The ideal dengue vaccine would produce rapid and highly protective, long-term, type- or cross-specific neutralizing antibodies against all DENV serotypes regardless of individual immune status and age of vaccination [170,238]. To date, no trialed dengue vaccine has achieves these criteria due to several major obstacles: (a) DENV evolution is rapid and unpredictable, generating many strains within DENV serotypes and (b) Great genetic variation either between the different serotypes or between viral genotypes within each serotype [238]. It is therefore very challenging to produce a vaccine that can induce a balanced and efficient immune response against all different genotypes of all serotypes. In short, a safe and efficacious vaccine continues to be sought internationally and the development of a safe, cross-protective and efficient vaccine, that does not induce antibody-dependent enhancement, is urgently required.

## 12. Conclusions and Future Perspectives

Dengue is the most important mosquito-borne viral disease in humans. It is a major public health concern in developing countries in Asia and Latin America. More detailed molecular epidemiological data are needed particularly from those regions where the data of circulating DENV are not available. Such information is important not only to predict dengue outbreaks but also for the consideration of vaccine design and composition. Conducting molecular epidemiological studies, including whole genome sequencing (WGS), is important in order to provide information on currently circulating viruses and to provide a better understanding of DENV transmission and epidemiology in a specific region. In regions where DENV co-circulating with other arboviruses such as such CHIKV and ZIKV, prospective cohort studies that assessing those viruses will provide an informative picture of the transmission dynamics of these arboviruses. More studies to investigate dengue pathogenesis are required, and the role of previous ZIKV infection on clinical outcomes should be further studied.

## Figures and Tables

**Table 1:** Description of the structural and non-structural proteins of dengue virus (DENV).

Protein	Structure	Function(s)
C	A 100 amino acid (aa) residue, homodimeric protein, containing four α-helical regions and an intrinsically disordered N-terminal domain.	C is involved in genome encapsidation.
prM/M	prM is a 166 aa residue protein, while M consists of 75 aa residues.	prM/M functions as a cap-like structure that protect the fusion peptide E from undergoing pre-mature fusion before virus release.
E	A 493–495 aa residue (53 kDa) class II N-glycosylated dimeric membrane fusion protein. In mature DENV, E is found as 90 homodimers that lie flat against the DENV surface forming a ‘smooth’ protein shell. Each of the monomer subunits is composed of three distinct domains (I, II and III).	E mediates virus binding and fusion to host cell membrane (domain III) and is responsible for the determination of host range, tropism and virulence (domain III).
NS1	A 46 kDa dimeric glycosyl-phosphatidylinositol (GPI) anchored protein that exists in both intra- and extracellular forms.	NS1 is involved in the viral RNA replication complex as well as in viral defense through the inhibition of complement activation.
NS2A	A 218 aa residue protein, 22 kDa.	NS2A involved in the coordination of change between RNA packaging and replication and antagonism of interferon (IFN).
NS2B	A 130 aa residue (14 kDa) protein and is a membrane-associated protein.	NS2B associates with NS3 to form the DENV protease complex and serves as a cofactor in the structural activation of the DENV serine protease of NS3.
NS3	A 618 aa (70 kDa) protein. The protease domain is N-terminal (residues 1–180) while the helicase domain is located at residues 180–618.	NS3 is multifunctional protein with chymotrypsin-like serine protease, RNA helicase, and RNA triphosphatase (RTP/NTPase) enzyme activity. NS3 is involved in cleaving the DENV polyprotein as well as RNA replication.
NS4A and NS4B	NS4A and NS4B are small hydrophobic proteins that consist 150 aa (16kDa) and 245–249 aa (27kDa), respectively. Both of them are integral membrane proteins.	NS4A induces membrane alterations that are important for virus replication. NS4B assists viral RNA replication through its direct interaction with NS3 and blocks IFN-induced signal transduction.
NS5	A 900 aa residues (104 kDa) protein and is the most conserved DENV protein. The methyltransferase domain is located at residues 1–269 while the RNA-dependent RNA polymerase is located at residues 270–900.	NS5 is a bifunctional enzyme with a methyltransferase and RNA-dependent RNA polymerase activity.

Sources: [11,12,18,19].

**Table 2:** Factors associated with dengue incidence or dengue outbreaks.

Factors	Reference(s)
Irregular use of vector control agent (temephos)	[38]
Lack of knowledge about dengue fever	[39]
Higher container index and higher Breteau index	[40]
Lower-altitude cantons, higher temperature, higher humidity and higher rainfall	[40,41,42]
Increased age	[39,43,44]
Housing located in close proximity to the markets, slum areas, or uncovered sewer areas	[38,45,46]
The presence of mosquito breeding sites in the garden or courtyard including the presence of discarded cans, discarded plastic containers, discarded tire casings, ponds, temples, receptacles in the plants with temporary water pools, gutter to collect rainwater, uncovered water storage container and food or water pans for animals.	[38,47,48,49,50]
Unscreened houses, high number of persons per room, inappropriate shower facilities, lack of waste collection, poor household water storage, lack of air-conditioning, and discharging sewage directly into ponds or street drainage	[39,43,44,45,46,48,51,52,53]

**Table 3:** Classification of DENV genotypes and their distributions.

Serotype	Genotype	Distribution
DENV-1	I	Strains from Southeast Asia, China and East Africa
	II	Strains from Thailand (between 1950s and 1960s)
	III	Sylvatic strain collected in Malaysia and Malaysian strain 36,046 (2005)
	IV	Strains from the West Pacific islands and Australia
	V	Strains collected in the Americas, West Africa, and some strains from Asia
DENV-2	Asian 1	Strains from Malaysia and Thailand
	Asian 2	Strains from Vietnam, China, Taiwan, Sri Lanka and the Philippines
	Cosmopolitan	Strains from Australia, East and West Africa, the Pacific and Indian ocean islands, the Indian subcontinent and the Middle East
	American	Strains from Latin America and older strains collected from the Caribbean, the Indian subcontinent and Pacific Islands in the 1950s and 1960s
	Asian/American	Strains from Thailand and Vietnam and strains collected in the Americas
	Sylvatic	Strains collected from humans, forest mosquitoes or sentinel monkeys in West Africa and Southeast Asia
DENV-3	I	Strains from Indonesia, Malaysia, the Philippines and recent isolates from the South Pacific islands
	II	Strains from Thailand, Vietnam and Bangladesh
	III	Strains from Sri Lanka, India, Africa and Samoa and 1962 strain from Thailand
	IV	Strains from Puerto Rico, Latin and Central America and the 1965 Tahiti strain
DENV-4	I	Strains from Thailand, the Philippines, Sri Lanka, and Japan
	II	Strains from Indonesia, Malaysia, Tahiti, the Caribbean and the Americas
	III	Thai strains that are distinct from other Thai isolates
	IV	Sylvatic strains from Malaysia

Sources: [18,35].

**Table 4:** Factors associated with severity of dengue.

Factor	Reference(s)
Demographics	
Children aged 6–10 years and girls	[182]
Caucasian race	[183]
Chinese ethnicity	[184]
Older age	[185]
Age >5 years or age >40 years	[186,187]
Female	[184,188,189]
Type of infection	
Secondary dengue infection	[139,187,190,191,192,193]
Laboratory parameters	
High hematocrit level	[188]
High alanine aminotransferase	[188]
Early increasing D-dimer	[194]
High cholesterol level	[195]
Low total serum cholesterol	[196]
Low-density lipoprotein C	[196]
Leucopenia	[186]
Hypoalbuminemia or hypoproteinemia	[189]
High alanine transaminase levels and aspartate transaminase, thrombocytopenia, prothrombin time, activated partial thromboplastin time and fibrinogen level	[189]
High hematocrit level	[197,198]
High lactate and absolute atypical lymphocyte levels.	[199]
Low platelet count	[198]
Thrombocytopenia	[200]
Clinical findings	
Mean arterial pressure <80 mmHg	[188]
Spontaneous bleeding	[186,189,198,200]
Hepatomegaly	[186,198,200]
Ascites	[186,189,198]
Pleural effusion	[186,189,198]
Persistent nausea/vomiting	[189,198,199]
Lethargy, thick gallbladder	[187,200]
Abdominal pain	[189,198,200]
Hemoconcentration	[200]
Headache, palmar erythema, joint pain, lymphadenopathy, giddiness, gallbladder wall edema	[198]
Hepato-splenomegaly	[189,198]
Hemoconcentration	[189]
Oliguria	[198]
Neurological signs	[189]
Virology	
DENV serotype (DENV-3 in primary infection in Southeast Asia)	[181]
High early viral load	[191]
Host factors	
Polymorphism in TNF-α, FcγRII, CTLA-4, TGF-β1, HPA, DC-SIGN, TAP, MBL2 and JAK1 gene.	[172]
Allergy treated with steroids and hypertension	[183]
ABO blood group	[190]
Diabetes	[201]
Hypertension (or with diabetes)	[184,202]
Skin allergy	[202]
Malnutrition (protective rule)	[203]

**Table 5:** Factors associated with mortality or fatality of dengue case.

Factors	Reference(s)
Demographic	
Female	[182]
Caucasoid race (compared to Negroid race, in Cuba)	[204]
Type of DENV infection	
Secondary DENV-2 infections	[205]
Clinical findings	
Persistent vomiting, lethargy, respiratory distress.	[206,207,208]
Plasma leakage (shock)	[206,209,210]
Severe bleeding (hemoptysis), epistaxis	[206,207,210]
Altered sensorium, abnormal reflexes, edema	[211]
Co-morbidities (diabetes mellitus and hypertension).	[211]
Myalgia	[212]
Had severe hepatitis, an altered mental status	[210]
Laboratory parameters	
High leucocyte counts	[208,210,212]
High platelet counts	[212]
High blood urea nitrogen or high creatinine level	[210,212]
Low serum albumin concentrations	[208]
High serum sodium, potassium, and bilirubin levels	[212]
High activated partial thromboplastin time, bilirubin levels and serum glutamic-pyruvic transaminase	[210]

**Table 6:** Summary of trial studies on dengue treatment.

Component	Dose(s)	Group Arm (T/P) *	Endpoint(s)	Main Finding(s)	Reference
Carbazochrome sodium sulfonate	300 mg infusion (day 1 and 2), followed by 150 mg (day 3)	Children 45/50	Primary: Reduction in number of patients experiencing shock or pleural effusion	There was no significant difference in shock or pleural effusion between groups	[227]
Chloroquine	600 mg loading dose followed by 600 mg (day 2) and 300mg (day 3) per oral	Adult 153/154	Primary: Time to resolution of viremia and NS1 antigenemia Secondary: Fever clearance time (FCT), the mean maximum% hemoconcentration and the proportion treated with intravenous fluid, became DHF, experienced with grade 3 or 4 adverse events, experienced vomiting and required blood transfusion.	There was no significant difference in primary endpoints between groups. Chloroquine was associated with reduction in FCT (intention-to-treat only), significantly less people developed DHF and vomiting. There was no significant finding for other secondary endpoints.	[232]
Prednisolone	(1). 2 mg/kg orally for 3 days (2). 0.5 mg/kg orally for 3 days	Children and young adults (1) 75/75 (2) 75/75	Primary: Clinical: The proportion of patients experiencing development of DSS or need for ICU admission and clinical bleeding. Hematological: Hyperglycemia, the platelet nadir, the maximum hematocrit between day 3 and day 8. Virological: Viremia and the viremia clearance time.	Hyperglycemia was higher in treatment groups (2 mg/kg). There was no significant findings of pre-defined clinical, hematological, or virological endpoints for both dose groups.	[228]
Balapiravir	(1). 3000 mg orally for 5 days (2). 1500 mg orally for 5 days	Adult (1) 22/32 (2) 10/32	Primary: Clinical signs and routine laboratory markers, plasma concentrations of 10 immune response components and viremia	There was no significant difference in all primary endpoints between groups.	[233]
Recombinant human IL-11	1.5 mg subcutaneously single dose	Adult 20/20	Primary: Increase in platelet count at 48 h	The increase of platelets in patients with severe thrombocytopenia was greater in the treatment group	[231]
Single donor platelet transfusion	Approximately 5 × 10^11^ platelets	Adult 43/44	Primary: Platelet count increments at 24 and 72 h. Secondary: Clinical parameters and AEs	The primary endpoints were significantly higher in the treatment group. Platelet transfusion did not prevent severe bleeding or shorten time to cessation of bleeding. Single donor platelet transfusion was associated with significant AEs.	[230]
Celgosivir	400 mg loading dose followed by 200 mg every 12 h orally (total nine doses)	Adult 24/26	Primary: Mean virological log reduction from baseline for days 2, 3, and 4, and area under the curve for a temperature above 37 °C from 0 h to 96 h. Secondary: AEs, hematological markers, NS1 clearance time	There was no significant difference in the primary endpoints between groups. There is no significant difference in AEs and hematological markers between groups but NS1 clearance time was substantially shorter in the treatment group.	[234]
Lovastatin	40 mg for 5 days orally	Adult 14/16	Primary: Safety	There was no difference in incidence of AEs between groups	[239]
Lovastatin	80 mg for 5 days orally	Adult 149/151	Primary: Safety Secondary: Disease progression rates, fever clearance time, and measures of plasma viremia and quality of life.	No difference in AEs and serious AEs observed between groups. There was no difference between groups of any secondary endpoints.	[240]

* T/P: treatment/placebo; AE: Adverse event, DHF: dengue hemorrhagic fever, DSS: dengue shock syndrome, ICU: intensive care unit, NS1: non-structural protein 1.
